# RETRACTION: Renal Function as Risk Factor for Diabetic Foot Ulcers: A Meta‐Analysis

**DOI:** 10.1111/iwj.70510

**Published:** 2025-04-18

## Abstract

**RETRACTION:** JinL. and XuW. , “Renal Function as Risk Factor for Diabetic Foot Ulcers: A Meta‐Analysis,” International Wound Journal 21, no. 3 (2024): e14409, 10.1111/iwj.14409.37991106 PMC10898369

The above article, published online on 22 November 2023, in Wiley Online Library (http://onlinelibrary.wiley.com/), has been retracted by agreement between the journal Editor in Chief, Professor Keith Harding; and John Wiley & Sons Ltd. Following an investigation by the publisher, all parties have concluded that this article was accepted solely on the basis of a compromised peer review process. The editors have therefore decided to retract the article. The authors did not respond to our notice regarding the retraction.

**RETRACTION:** L. Jin and W. Xu , “Renal Function as Risk Factor for Diabetic Foot Ulcers: A Meta‐Analysis,” International Wound Journal 21, no. 3 (2024): e14409, 10.1111/iwj.14409.37991106 PMC10898369

The above article, published online on 22 November 2023, in Wiley Online Library (http://onlinelibrary.wiley.com/), has been retracted by agreement between the journal Editor in Chief, Professor Keith Harding; and John Wiley & Sons Ltd. Following an investigation by the publisher, all parties have concluded that this article was accepted solely on the basis of a compromised peer review process. The editors have therefore decided to retract the article. The authors did not respond to our notice regarding the retraction.

